# Aspirin Use and Mortality in Women With Ovarian Cancer: A Meta-Analysis

**DOI:** 10.3389/fonc.2020.575831

**Published:** 2021-02-01

**Authors:** Xiaxia Man, Baogang Wang, Yuying Tan, Xiaolin Yang, Songling Zhang

**Affiliations:** ^1^ Department of Oncological Gynecology, First Hospital, Jilin University, Changchun, China; ^2^ Department of Cardiac Surgery, First Hospital, Jilin University, Changchun, China; ^3^ Department of Echocardiography, First Hospital, Jilin University, Changchun, China; ^4^ Department of Geriatrics, First Hospital, Jilin University, Changchun, China

**Keywords:** aspirin, ovarian carcinoma, mortality, cohort studies, meta-analysis

## Abstract

**Background:**

Aspirin use has been suggested to reduce the incidence of ovarian cancer (OC) in women. However, previous studies regarding the association between aspirin use and mortality in women with OC showed inconsistent results. We aimed to evaluate the association between aspirin use and mortality in women with OC in a meta-analysis.

**Methods:**

Relevant cohort studies were obtained *via* search of PubMed, Cochrane’s Library, and Embase databases from inception to May 3, 2020. A random-effect model, which incorporates the potential heterogeneity among the included studies, was used to pool the results. Predefined stratified analyses were applied to evaluate the potential study characteristics on the outcome, including the timing of aspirin use, dose of aspirin, age of the women, and the clinical stages of the cancer. Sensitivity analysis by omitting one study at a time was used to assess the stability of the results.

**Results:**

Six cohort studies including 17,981 women with OC were included. Pooled results showed that aspirin use had no statistically significant association with mortality in these patients (adjusted risk ratio [RR]: 0.85, 95% confidence interval [CI]: 0.70 to 1.02, p = 0.08; I^2^ = 69%). The results were similar for OC-specific mortality (RR: 0.85, 95% CI: 0.57 to 1.26, p = 0.41) and all-cause mortality (RR: 0.78, 95% CI: 0.55 to 1.11, p = 0.17). Stratified analyses suggested that aspirin use had no statistically significant association with mortality risk in OC regardless the timing of aspirin use, dose of aspirin, age of the women, or the clinical stages of the cancer. Funnel plots suggested potential risk of publication bias (p all > 0.05). However, further “trim-and-fill” analysis incorporating hypothesized unpolished studies to achieve symmetrical funnel plots showed similar results of the meta-analysis (RR: 0.91, 95% CI: 0.74 to 1.13, p = 0.39).

**Conclusions:**

Current evidence from observational studies indicated that aspirin use had no statistically significant association with mortality in women with OC.

## Introduction

Ovarian cancer (OC) is the seventh most commonly diagnosed malignancies and the eighth leading cause of cancer-specific mortality for women all over the world (1, 2). Due to the lack of effective screening protocol for early detection of OC on a population level, substantial women with symptoms related with OC are diagnosed at advanced stages (3, 4). Accordingly, the overall 5-year survival for women diagnosed with OC is less than 50% (5). Therefore, development of strategies to reduce the mortality in women with OC remains of clinical significance (6, 7). Accumulating evidence from epidemiological studies suggests that aspirin, one of the most commonly used non-steroidal anti-inflammatory drugs (NSAIDs) (8), may have anticancer efficacy (9). Via irreversible inactivation of cyclooxygenase (COX), aspirin is suggested to exert antiplatelet and anti-inflammatory efficacies (8), which have been considered to inhibit the carcinogenesis and metastasis of tumors (10). In addition, observational studies demonstrated that aspirin use is likely to be associated with reduced incidence of many cancers, such as endometrial cancer (11), gastric cancer (12), colorectal and other digestive tract cancers (13), pancreatic cancer (14), hepatocellular carcinoma (15), and OC (16–18). However, uncertainty remains regarding the influence of aspirin use on mortality in women with OC, and previous observational studies evaluating this association showed inconsistent results (19). Some studies suggested that aspirin use was associated with reduced mortality events in women with OC (20–22), while others did not (23–25). In this study, we performed a meta-analysis to systematically evaluate the influence of aspirin use on mortality in women with OC.

## Methods

The meta-analysis was performed in accordance with the MOOSE (Meta-analysis of Observational Studies in Epidemiology) (26) and Cochrane’s Handbook (27) guidelines.

### Literature Search

Studies were identified *via* systematic search of electronic databases of PubMed Cochrane’s Library, and Embase *via* the following terms: (1) “aspirin” OR “antiplatelet” OR “non-steroidal anti-inflammatory drugs” OR “NSAIDs”; (2) “ovarian” OR “ovary”; (3) “cancer” OR “carcinoma” OR “tumor” OR “malignancy” OR “neoplasm” and (4) “mortality” OR “death” OR “survival” OR “recurrence” OR “prognosis” OR “progression”). The extensive search strategy was used for database search to avoid missing any possibly related studies. The search was limited to human studies without language restriction. The reference lists of related original and review articles were also analyzed using a manual approach. The final literature search was performed on May 3, 2020.

### Study Selection

The inclusion criteria for the studies were: (1) longitudinal follow-up studies including randomized controlled trials (RCTs), cohort studies, and nested case–control studies, which were published as full-length articles; (2) women with confirmed diagnosis of OC at baseline were included; (3) association between aspirin use and mortality events during follow-up, including OC-specific mortality and/or all-cause mortality, was evaluated; (4) with a minimal follow-up duration of one year; and (5) reported the relative risk for this association after adjustment of potential confounding factors at least of age. Reviews, editorials, preclinical studies, and studies irrelevant to the aim of current meta-analysis were excluded.

### Data Extracting and Quality Evaluation

Literature search, data extraction, and quality assessment of the included studies were independently performed by two authors according to the predefined criteria. Discrepancies were resolved by consensus or discussion with the corresponding author. Following data were extracted: (1) name of first author, publication year, and country where the study was performed; (2) study design characteristics; (3) patient characteristics, including sample size, age, and clinical stage of OC; (4) definition of aspirin use; (5) follow-up durations; (6) outcomes reported; and (7) confounding factors that were included in the multivariate analyses. The quality of each study was evaluated using the Newcastle-Ottawa Scale (28) which ranges from 1 to 9 stars and judges each study regarding three aspects: selection of the study groups; the comparability of the groups; and the ascertainment of the outcome of interest.

### Statistical Analyses

We used risk ratios (RRs) and their corresponding 95% confidence intervals (CIs) as the general measure for the association between aspirin use and clinical outcomes. Data of RRs and their corresponding stand errors (SEs) were calculated from 95% CIs or p values, and were logarithmically transformed to stabilize variance and normalized the distribution (27). The Cochrane’s Q test and estimation of I^2^ statistic were used to evaluate the heterogeneity among the include cohort studies (29). A significant heterogeneity was considered if I^2^ >50%. We used a random-effect model to synthesize the RR data because this model is considered as a more generalized method which incorporates the potential heterogeneity among the included studies (27). Sensitivity analyses by omitting one individual study at a time were performed to test the robustness of the results (30). Stratified analyses were performed according to the definition of mortality outcome, timing of aspirin use, aspirin dose, age of the women, and the International Federation of Gynecology and Obstetrics (FIGO) stage of OC. The potential publication bias was assessed by funnel plots with the Egger’s regression asymmetry test (31). A “trim-and-fill” analysis was performed if publication bias was suggested. By incorporating the imputed unpublished studies, symmetrical funnel plots were generated *via* this method and the results of pooled analysis including these hypothesized studies were subsequently performed (27). We used the RevMan (Version 5.1; Cochrane Collaboration, Oxford, UK) and STATA software for the meta-analysis and statistics.

## Results

### Literature Search

**Figure 1:**
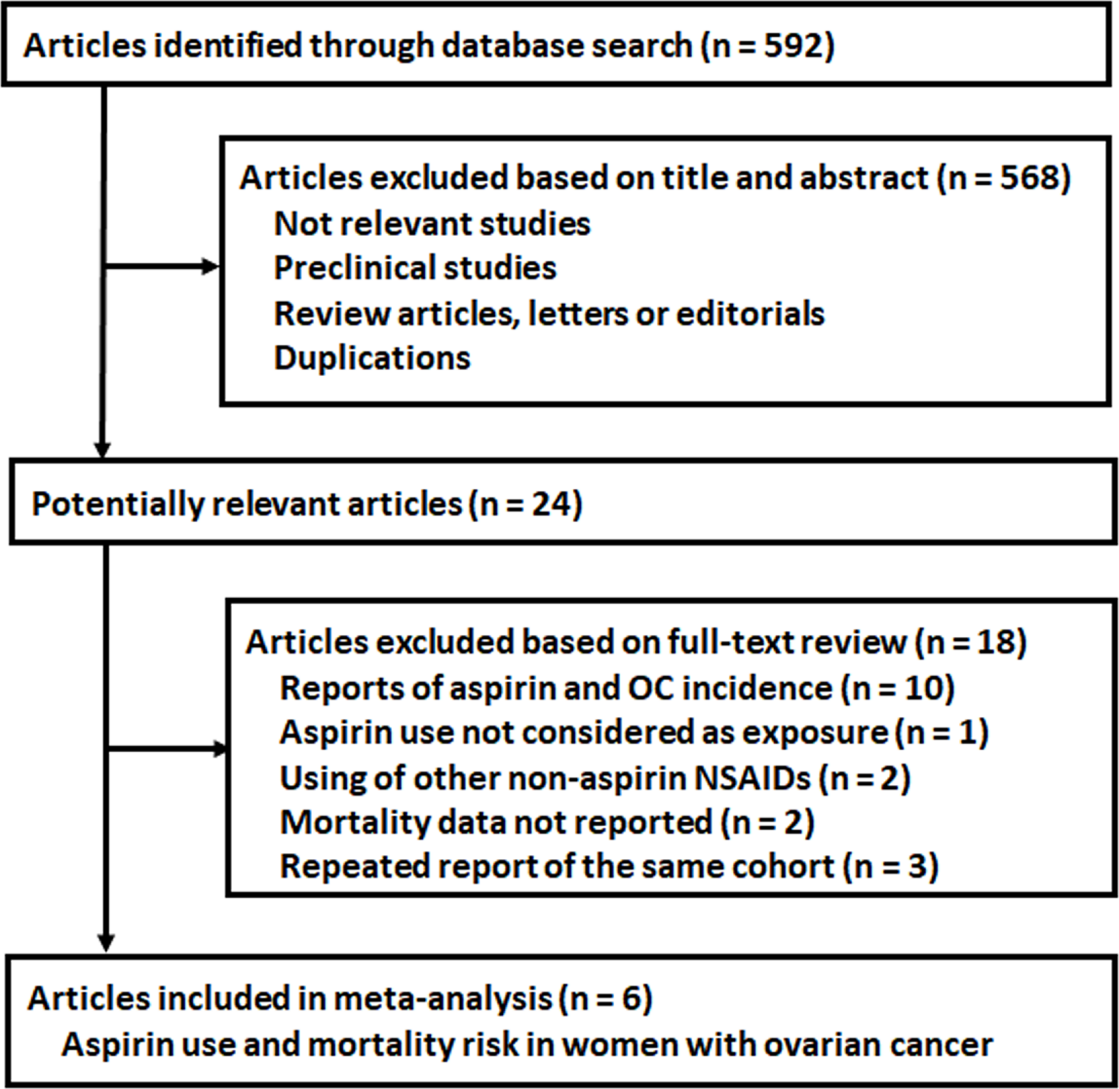
Flowchart of literature search.

The process of database search was summarized in **Figure 1**. Briefly, 592 articles were found *via* initial literature search of the PubMed, Cochrane’s Library, and Embase databases, and 568 were excluded through screening of the titles and abstracts mainly because they were not relevant to the purpose of the meta-analysis. Subsequently, 24 potential relevant records underwent full-text review. Of these, 18 were further excluded based on reasons listed in **Figure 1**. Finally, six studies were included (20–25).

### Study Characteristics and Quality Evaluation

The characteristics of the included studies were summarized in **Table 1**. One study was a pooled analysis of individual-level data from 12 Ovarian Cancer Association Consortium studies (23), four were retrospective cohort studies (20, 22, 24, 25), and the other one was a prospective cohort study (21). Overall, 17,981 women with confirmed diagnosis of OC from Israel, USA, UK, Australia, and Denmark were included in these studies. The sample sizes of the included women varied between 77 and 11,967. The mean age of the included women varied from 53 to 64 years. Four studies included women with FIGO stages I–IV OC (20, 22–24), while the other two studies included women with stages I–III (21) and IIIC–IV (25) OC, respectively. Aspirin users were validated by prescription records in five studies (20–22, 24, 25), and by self-report questionnaires in one study (23). Moreover, post-diagnostic use of aspirin was analyzed for the included studies, except for one study in which pre-diagnostic use of aspirin was analyzed (23). The follow-up duration varied from 2.1 to 7.8 years. Potential confounding factors, such as age, cancer stages, anticancer treatments, comorbidities, and other concurrent medications, were adjusted in the multivariate analyses of the original studies. The NOS scores of the included studies ranged from six to nine, indicating generally good study quality (**Table 2**).

**Table 1:** Characteristics of the included cohort studies.

Study	Design	Country	Patient characteristics	Patient number	Mean age	FIGO stage	Definition of aspirin use	Follow-up duration	Outcomes reported	Outcome validation	Variables adjusted
					years			years			
Bar et al. (20)	RC	Israel	Women with OC treated by debulking surgery and platinum based chemotherapy	143	62.5	I–IV	Aspirin use after OC diagnosis by pharmacy records	2.1	All-cause deaths (78)	Medical database	Age, cancer stage, cancer treatment, comorbidities, and concurrent medications
Dixon et al. (23)	Pooled study of RC	USA, UK, Australia, and Denmark	Women with epithelial OC	11967	57.0	I–IV	Self-reported use of aspirin at least 1/w before OC diagnosis	7.3	All-cause deaths (4,273)	National death index	Age, education, and ethnicity
Verdoodt et al. (24)	RC	Denmark	Women aged 30–84 years with primary epithelial OC	4117	62.5	I–IV	Low-dose aspirin use after the diagnosis of OC	3.6	OC-specific deaths (2,245)	National demographic, prescription, and patient registries	Age, education, disposable income, marital status, comorbid, cancer stage, histology, chemotherapy, and non-aspirin drug use
Wield et al. (22)	RC	USA	Women with clear cell OC	77	53.4	I–IV	Aspirin use after the diagnosis of OC	7.8	All-cause deaths (33)	Medical database	Age, cancer stage and cytoreductive status
Merritt et al. (21)	PC	USA	Women with invasive epithelial OC	1143	63.2	I–III	Aspirin use after the diagnosis of OC within 2y	4.2	OC-specific deaths (491)	Medical database	Age, cancer stage, histology, parity, duration of oral contraceptive use, menopausal status, family history of OC, smoking, BMI, and concurrent medications
Gonzalez et al. (25)	RC	USA	Women with stage IIIC–IV epithelial OC	534	64.0	IIIC–IV	Aspirin use after the diagnosis of OC	6.2	All-cause deaths (339)	Medical database	Age, race, age, CCI, cancer stage, triage to neoadjuvant chemotherapy, histology, residual disease status, and concurrent medications

OC, ovarian cancer; FIGO, International Federation of Gynecology and Obstetrics; NOS, Newcastle-Ottawa Scale; RC, retrospective; PC, prospective; BMI, body mass index; CCI, Charlson Comorbidity Index.

**Table 2:** Details of study quality evaluation *via* the Newcastle-Ottawa Scale.

Study	Representativeness of the exposed cohort	Selection of the non-exposed cohort	Ascertainment of exposure	Outcome not present at baseline	Control for age and gender	Control for other confounding factors	Assessment of outcome	Enough long follow-up duration	Adequacy of follow-up of cohorts	Total
Bar et al. (20)	0	1	1	1	1	1	1	0	1	7
Dixon et al. (23)	1	1	0	1	1	1	0	1	1	7
Verdoodt et al. (24)	1	1	1	1	1	1	1	0	1	8
Wield et al. (22)	0	1	1	1	1	0	0	1	1	6
Merritt et al. (21)	1	1	1	1	1	1	1	1	1	9
Gonzalez et al. (25)	0	1	1	1	1	1	0	1	1	7

### Meta-Analysis Results

**Figure 2:**
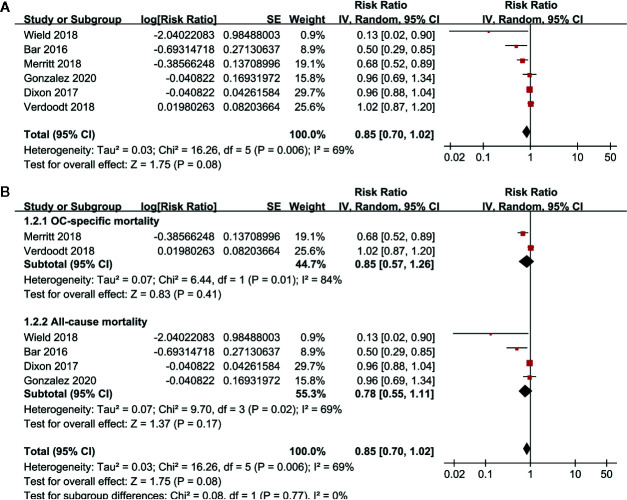
Forest plots for the meta-analysis of the association between aspirin use and mortality risk in women with OC; **(A)** overall meta-analysis; and **(B)** subgroup analysis by the definition of mortality outcome.

**Figure 3:**
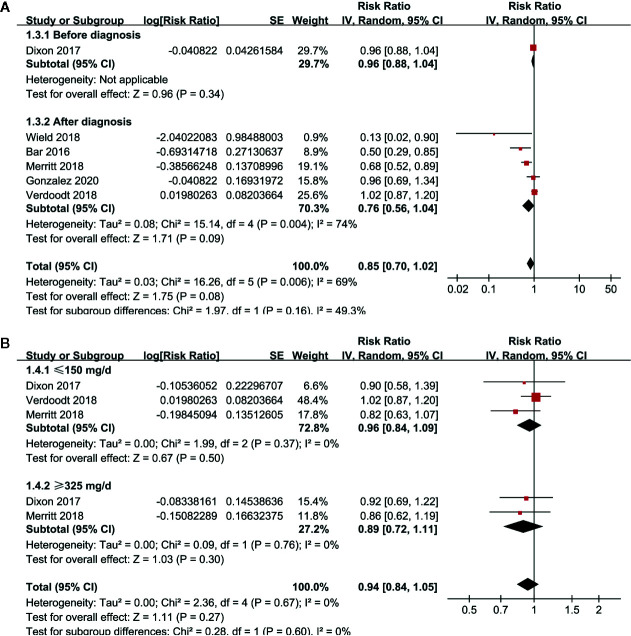
Stratified analyses for the meta-analysis of the association between aspirin use and mortality risk in women with OC; **(A)** stratified according to the timing of aspirin use; and **(B)** stratified according to the dose of aspirin.

**Figure 4:**
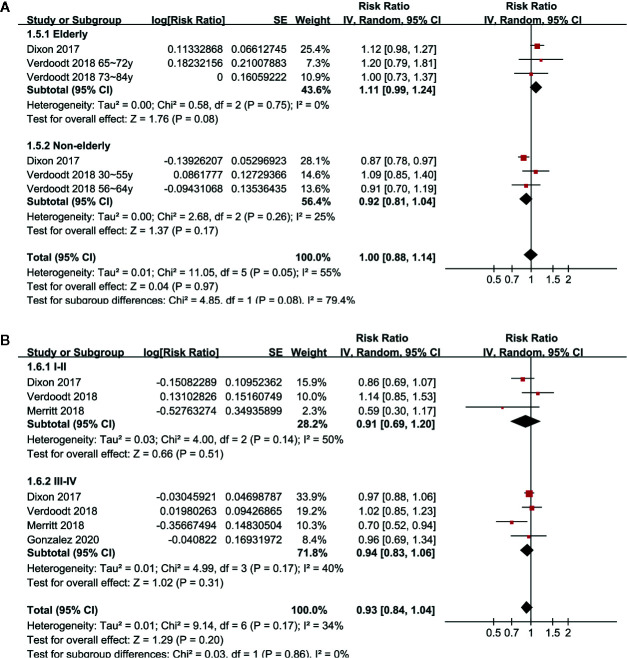
Stratified analyses for the meta-analysis of the association between aspirin use and mortality risk in women with OC; **(A)** stratified according to the age of the women; and **(B)** stratified according to the FIGO stage of OC.

Pooled results of six studies with a random-effect model showed that aspirin use had no statistically significant association with mortality in these patients (adjusted RR: 0.85, 95% CI: 0.70 to 1.02, p = 0.08) with significant heterogeneity (p for Cochrane’s Q test = 0.006, I^2^ = 69%; **Figure 2A**). Sensitivity analysis by omitting either of the included study did not significantly change the results (RR: 0.76 to 0.90, p all > 0.05; **Table 3**). Subgroup analysis showed similar results in studies that reported OC-specific mortality (RR: 0.85, 95% CI: 0.57 to 1.26, p = 0.41) and all-cause mortality (RR: 0.78, 95% CI: 0.55 to 1.11, p = 0.17; **Figure 2B**). Further stratified analyses showed that aspirin use had no statistically significant association with mortality in women with OC in studies of aspirin use before (RR: 0.96, 95% CI: 0.88 to 1.04, p = 0.34) and after the diagnosis of OC (RR = 0.76, 95% CI: 0.56 to 1.04, p = 0.09; **Figure 3A**), in studies with aspirin dose ≤ 150 mg/day (RR: 0.96, 95% CI: 0.84 to 1.09, p = 0.50) and ≥ 325 mg/day (RR = 0.89, 95% CI: 0.72 to 1.11, p = 0.30; **Figure 3B**), in elderly (RR: 1.11, 95% CI: 0.99 to 1.24, p = 0.08) and non-elderly women with OC (RR = 0.92, 95% CI: 0.81 to 1.04, p = 0.17; **Figure 4A**), and in women with stages I–II (RR: 0.91, 95% CI: 0.69 to 1.20, p = 0.51) and stages III–IV OC (RR = 0.94, 95% CI: 0.83 to 1.06, p = 0.31; **Figure 4B**).

**Table 3:** Results of sensitivity analysis.

Studies excluded	RR	95% CI	I^2^	P for effect
Baret al. (20)	0.90	0.76 to 1.06	63%	0.22
Dixon et al. (23)	0.76	0.56 to 1.04	74%	0.09
Verdoodt et al. (24)	0.76	0.58 to 1.02	73%	0.07
Wield et al. (22)	0.87	0.73 to 1.03	67%	0.10
Merritt et al. (21)	0.90	0.75 to 1.09	62%	0.29
Gonzalez et al. (25)	0.81	0.65 to 1.02	75%	0.08

RR, risk ratio; CI, confidence interval.

### Publication Bias

**Figure 5:**
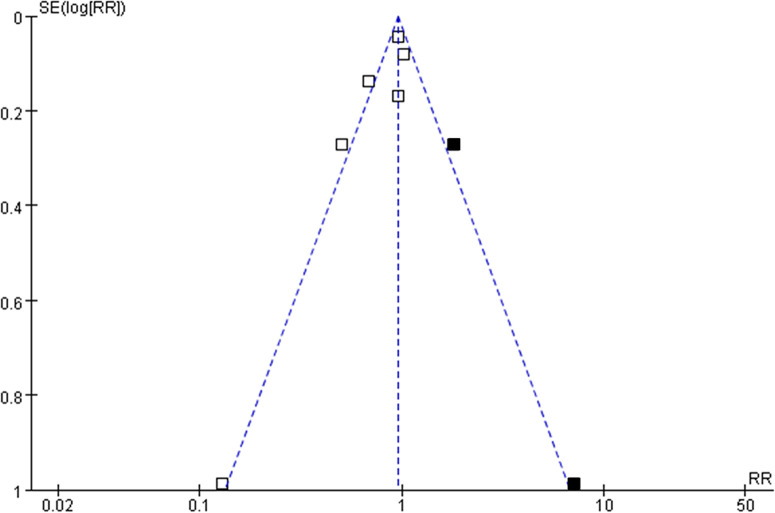
Funnel plots with “trim-and-fill” analysis for the publication bias of the meta-analysis of the association between aspirin use and mortality risk in women with OC; the black squares indicated the hypothesized studies, which were also incorporated to generate symmetrical funnel plots.

The funnel plots for the meta-analysis of the association between aspirin use and mortality risk in women with OC were shown in **Figure 5**. The plots were asymmetrical on visual inspection, suggesting potential risks of publication biases. By trim-and-fill analyses, two hypothesized studies were imputed. Meta-analysis incorporating these two studies did not significantly change the results (RR: 0.91, 95% CI: 0.74 to 1.13, p = 0.39).

## Discussion

In this meta-analysis of cohort studies, we found that aspirin use had no statistically significant association with mortality risk in women with OC. Sensitivity analyses by excluding one study at a time did not change the results, indicating that the results of the meta-analysis were stable. Subgroup analyses showed consistent results in studies reporting OC-specific mortality or all-cause mortality. Stratified analyses showed that aspirin use had no statistically significant association with mortality in women with OC regardless the timing of aspirin use, dose of aspirin, age of the women, or the clinical stages of the cancer. Taken together, these results suggested that based on current evidence from epidemiological studies, aspirin use had no statistically significant association with mortality in women with OC.

A previous pooled analysis of individual-level data from 12 Ovarian Cancer Association Consortium studies showed that in women with OC, self-reported pre-diagnostic use of aspirin was not associated with significantly affected all-cause mortality (23). Our meta-analysis based on study-level further confirmed that aspirin use was not associated with significantly affected mortality risk in these patients. Both retrospective and prospective cohort studies were included. Moreover, aspirin use was validated by pharmacy records in five of the included studies (20–22, 24, 25), which were less biased than self-reported use in questionnaires. Different from the previous pooled-analysis, we performed multiple stratified analyses to evaluate whether the association could be affected by factors such as the timing of aspirin use, dose of aspirin, age of the women, and the clinical stages of the cancer. All of the above stratified analyses showed no significant association between aspirin use and mortality of OC. These findings further confirmed that aspirin use had no statistically significant association with mortality in women with OC.

These findings are contrary to previous preclinical studies, which generally indicated that aspirin confers anticancer efficacy in OC. An early *in-vitro* study showed that aspirin inhibited ovarian tumor cell growth in a dose-dependent manner (32). Subsequent studies showed that aspirin could inhibit the proliferation of ovarian tumor cells *via* multiple mechanisms, such as direct inactivation of COX-2 (33) and inhibition of platelet activation (34). Besides, aspirin was shown to exert antiangiogenic effect in mice model of OC, suggesting its inhibitory effect on metastasis of ovarian tumor cells (35). The potential reasons for the discrepancies between the results of meta-analysis and preclinical studies may primarily be attributed to the complexity of the role of aspirin in women with OC than that in tumor cell lines or animal models. The comorbidities and concurrent medications are likely to affect the potential anticancer efficacy of aspirin. Moreover, the possible anticancer efficacy of aspirin may become clinically irrelevant in women who had already received adequate treatments against OC based on current medical recommendation. However, it has to be mentioned that it is somewhat biased to evaluate the potential efficacy of aspirin on survival in OC based on cohort studies. In these studies, aspirin was prescribed for other indications rather than the anticancer purpose. Therefore, those who use aspirin before the diagnosis of the cancer are likely to have other comorbidities which could lead to poor prognosis, thereby masking the potential benefits of aspirin. Moreover, those who have more advanced cancer, and thus a worse prognosis, might be less likely to take aspirin after diagnosis, which may be another reason to confound the results. Considering these potential biases, the efficacy of aspirin on mortality in women with OC should be optimally evaluated in clinical trials.

Our study has limitations. Firstly, studies available for the meta-analysis and stratified analyses were limited. Moreover, since we did not have access to individual-patient data of the included studies, the stratified analyses were performed based on study-level data. The findings should be interpreted with caution. Secondly, significant heterogeneity was found among the included studies. Although stratified analyses did not support that timing of aspirin use, dose of aspirin, age of the women, and the clinical stages of the cancer may contribute to the heterogeneity. We could not exclude other characteristics that may contribute to the heterogeneity, such as the histological subtype of OC and the anticancer treatments applied in each study. Further studies are needed to evaluate the influence of these factors on the outcomes. Finally, as mentioned above, due to the inherited biases in observational studies, RCTs are warranted to validate the efficacy of aspirin on mortality in women with OC.

In conclusion, results of the meta-analysis based on evidence from epidemiological studies showed that aspirin use had no statistically significant association with mortality in women with OC. In view of the consistent anticancer efficacy of aspirin observed in the preclinical studies, as well as the potential limitations of observational studies, clinical trials may be considered to investigate the influence of aspirin on mortality in women with OC.

## Data Availability Statement

The original contributions presented in the study are included in the article/supplementary material. Further inquiries can be directed to the corresponding authors.

## Author Contributions

XY and SZ conceived and designed the study. XM and BW selected the studies and collected the data. XM and YT analyzed data. All authors interpreted the results. XM, XY, and SZ drafted the paper. All authors revised the draft paper. All authors contributed to the article and approved the submitted version.

## Conflict of Interest

The authors declare that the research was conducted in the absence of any commercial or financial relationships that could be construed as a potential conflict of interest.
